# Cost and Quality Optimization Taguchi Design with Grey Relational Analysis of Halloysite Nanotube Hybrid Composite: CNC Machine Manufacturing

**DOI:** 10.3390/ma15228154

**Published:** 2022-11-17

**Authors:** Moses Olabhele Esangbedo, Johnson Kehinde Abifarin

**Affiliations:** 1 School of Management Engineering, Xuzhou University of Technology, No. 2 Lishui Road, Yunlong District, Xuzhou 221018, China; 2 Department of Mechanical Engineering, Ahmadu Bello University, Zaria 810211, Nigeria

**Keywords:** CNC machine, hybrid composite, grey relational analysis, modeling, surface roughness, depth of cut, feed rate

## Abstract

Researchers who work on manufacturing hybrid composites have significant concerns about holistically optimizing more than one performance characteristic, as in the case of cost and quality optimization. They usually trade off one for the other. Hence, this study employed statistical tools and grey relational analyses (GRA) design to model and optimize the surface roughness and cutting force of Computer Numerical Control (CNC) machine settings to manufacture halloysite nanotube hybrid composite. In this paper, the GRA was able to address the multiple optimization complications by producing 0.6 mm depth of cut, 1500 rpm spindle speed, and 40 mmpm feed rate as the CNC machine settings for high-quality and low-cost hybrid composite. It was noticed that the mathematical and interaction modeling of surface roughness, cutting force, and grey relational grade (GRG) allowed different CNC machines to manufacture hybrid composites. This can assist researchers and production engineers of CNC machines. Variance analysis and delta statistical characteristics revealed that the depth of a cut is the most significant machine setting, with a contribution of 49.12%. This paper outlines the possible CNC machine settings for high-quality composite manufacturing. In future studies, it is recommended for researchers in the field of CNC machine manufacturing to consider the modeling analysis aspect of the optimization, which comprehensively provides the opportunity for the adjustment of CNC machines for better material performance, which has been lacking in the literature.

## 1. Introduction

It has been extensively proven that hybrid composite structures have exceptional properties, such as low weight, high strength, and low cost, compared to their counterpart materials [1,2,3]. These make them suitable for several engineering applications. However, it is important to state that the production of hybrid composite materials is complex in terms of design for optimal and reliable tendencies. When the design process is not carefully considered, their production is costly, limiting their applications.

Epoxy resins are known for high performance. They are the building blocks for adhesives, coatings, reinforced plastics, and composite materials, such as fiberglass and carbon fiber, which remain intact under intense conditions. When properly cured, epoxy resins offer some desirable characteristics, such as resistance to chemicals, particularly in alkaline environments, heat resistance, adhesion to a variety of substrates, high tensile, compression, and bending strengths, low shrinkage during curing, high electrical insulation and retention properties, corrosion resistance, cures under a wide range of temperatures, and resistance to fatigue [4]. The properties of epoxy resins have drawn the attention of researchers and material production engineers to design and manufacture the material for different applications.

Halloysite nanotubes (HNTs) occur naturally with unique properties. Berthier described HNTs as a mineral in the Kaolin group [5]. HNTs are an aluminosilicate in a double-layered form with a composition of aluminum, hydrogen, oxygen, and silicon. It has a chemical composition of Al${}_{2}$Si${}_{2}$O${}_{5}$(OH)${}_{4}$·nH${}_{2}$O [6,7,8]. A halloysite nanocomposite is synthesized by hydrothermal carbonization of cellulose onto halloysite, forming a layer of carbon on the halloysite surface. HNTs in the composite are said to improve thermal stability, reduce crack growth, and improve the strength and toughness of epoxy composite [9,10,11,12]. Aluminum as a filler for the hybrid composite has been said to essentially improve material machinability [13,14]. The employed hybrid composite has remarkable potential in aerospace, tooling, automobile, and defense applications. Yuan et al. [15] summarized the properties of halloysite material as a one-dimensional mesoporosity or macroporosity, enabling the encapsulation of various active guests in the lumen of HNTs that acts as a nanoscale container, as well as the subsequent controlled release of the guests. The HNTs are also an effective nanofiller because HNTs are naturally dispersed, unlike the traditional. It is said that halloysite has a higher water content compared to its kaolinite counterpart [16].

In this study, Taguchi is employed to help with regression modeling and better optimization analysis. The grey system theory was introduced by Deng [17], which deals with systems with incomplete information, and there are several applications of the grey system theory [18,19]. Grey relational analysis (GRA) is employed to assist in the optimization of the considered engine parameters. It is employed to address the inadequacy of the Taguchi design technique, which can only optimize one performance characteristic. Specifically, this study addresses the optimization of two performance characteristics: the surface roughness of the manufactured hybrid composite and the cutting force of the employed machine. GRA helps in any complication developed due to different optimal settings that can be obtained from individual optimization of many performance characteristics [20]. Hence, this study employed Taguchi GRA to optimize and model machining parameters for cost minimization and quality maximization. The surface roughness of the manufactured composite is the quality function while cutting force is the cost function in this study.

This study builds on the work by Pang et al. [21]. They have laboriously conducted the experimental study, but their study is inadequate for efficient analysis, conclusion, and recommendation. This study does not involve experimental work but purely the employment of the aforementioned optimization and modeling tools. Microsoft Excel, Minitab 16, and Origin 2019 software were employed for analysis in this study. More importantly, this paper makes the following contributions. First, provide a better setting for CNC machine manufacturing. Secondly, provide the application of the GRA for halloysite nanotube hybrid composite development. The remainder of the paper is presented as follows: Section 2 presents the literature review section. Section 3 presents the materials and methods used in this research. Section 4 presents the results and discussion. The conclusion is drawn in Section 5.

## 2. Literature Review

### 2.1. Related Works and Findings

The GRA is a multi-criteria decision-making method with wide applications in natural science, social science, and engineering technology [22,23,24]. Much research has been conducted on the production optimization of hybrid composite materials. Pang et al. [21] fabricated a halloysite nanotube hybrid composite using the Taguchi design technique for machining experimental parameter layouts. Their study had an unrealistic consideration of singular optimization and was also largely deficient in deep optimization analysis in terms of modeling versus experimental analysis. Analysis of variance was a very important analytical technique when the Taguchi design method was used. However, it was not employed in their study. Although multi-response consideration was performed with quantitative effect analysis, a deeper analysis in terms of machining parameters interaction and modeling for composite output performance was not needed to be performed in their study. Kalantari et al. [25] decided to minimize the density and cost of producing an epoxy hybrid composite with varying fiber orientation, type, and volume fraction using the Pareto optimal solutions technique. Their results showed a proven cost minimization, but there was a tradeoff in composite quality because it was only optimized for a singular response.

In addition, Rao et al. [26] employed the elitist non-dominated sorting genetic algorithm for the optimization of jet machining parameters in the production of the epoxy hybrid composite. The Taguchi design technique was employed to investigate the composite output performance when the considered machining parameters interacted. The gap in their study was that there was no comparative study of the experimental data with the modeled data for substantial conclusions and recommendations. An et al. [27] maximized the fundamental frequency and minimized the material cost in the production of laminated hybrid composite using approximate multipoint functions and the genetic algorithm. It was found that modeling and interaction of optimal parameters were not reported. Material quality and parameter’s quantitative effect were also not reported for better and informed design knowledge. Ma et al. [28] employed a discrete material optimization technique to optimize the framework for hybrid composite production. It was found that their study was only concerned with the different cost components of the materials at the expense of material quality. Beylergil [29] employed a multi-objective genetic algorithm to optimize fiber properties for the production of glass–fiber hybrid composite. It was asserted that the optimization technique employed was efficient for the hybrid composite structural performance; it was observed in their study that the technique is inadequate for the optimization of other salient properties of the hybrid composite. The quantitative effect of the framework on the resultant material cost was not figured out. Kayaroganam et al. [30] employed a non-dominated sorting genetic algorithm and statistical analysis for the optimization of drilling parameters in the production of a metal hybrid composite. Despite multi-response consideration, their work did not study drilling parameter interaction for better material performance. Also, the quantitative and qualitative effects of drilling parameters were not put into consideration. Kumar et al. [31] employed an analytical hierarchy process and genetic algorithm to select optimal machining parameters for the production of an aluminum metal matrix hybrid composite. In their work, multi-response optimization was considered, but the relationship between different parameters for the best material performance was not considered, which can make the maneuvering of the available machining parameters not easy.

Furthermore, Kandasamy et al. [32] minimized the erosion rate of friction-stirred fabricated aluminum hybrid composite using the Taguchi design technique. Erosion rate as a cost component is not the only essential component because the material quality in terms of finishing and machining efficiency is important, which subsequently gives a holistic view of the production cost analysis. Peng et al. [33] employed an adaptive non-dominated sorting genetic algorithm to optimize the production parameters of laminated hybrid composite. The issue of modeling and the interaction of optimal parameters were not conducted in their study. González et al. [34] employed grinding flank tools to carry out super abrasive machining of integral rotary components. Obviously, their work made an in-depth study on cutting force and surface roughness of the machined components. However, clearly, there was no consideration of performance optimization whatsoever. This was a shortcoming in drawing a clearer and more conclusive recommendation. Kondayya and Krishna [35] modeled and optimized the CNC end-milling process using an integrated evolutionary technique. Although they used the same machine conditions as the present study, the performance responses (material removal rate and tool wear) considered in their work were different from those considered in the present study, which are cutting force and surface roughness. Ribeiro et al. [36] optimized the cutting parameters and minimized the surface roughness of the end-milling process with the help of the Taguchi design technique. In their study, only a singular optimization analysis was performed, which is the main drawback. Multi-response optimization analysis was essential in their research because they evaluated more than one performance response, and the general performance of the milling process is significant. Achuthamenon Sylajakumari et al. [37] conducted multi-response optimization on the wear rate and coefficient of friction of co-continuous composite using Taguchi GRA. Without mincing words, their study presented a multi-response optimization analysis. However, the study was deficient in mathematical and graphical modeling optimization analysis. This analysis was crucial for accommodating optimization in the case that there are varying machine conditions. Undoubtedly, there is room to address the limitation of these studies.

### 2.2. Taguchi Singular and Multi-Response Optimization Survey

It is essential to bring to the limelight the literature on cost and quality optimization in manufacturing hybrid composites. Proper management of machining parameters for effective production and low cost of production is critical for any product or composite development. This study will assist in determining cost and quality optimization for the sample composite and any typical composite or product when machining is used for production. The Taguchi design technique is employed in this study to reduce the cumbersome nature of the production and to have a proper and in-depth analysis [22,38]. The Taguchi design helps manufactured products meet consumers’ expectations. It integrates the quality of the product in the design because the process of manufacturing should not be performed at the expense of product or process quality. Korucu et al. [39] employed TOPSIS with the assistance of the Taguchi design to optimize synthesized graphene oxide powders. Park et al. [40] employed the Taguchi design technique to determine the maximum and robust voltage within a wide range of frequencies of a piezoelectric cantilever beam. Obiko et al. [41] employed the Taguchi design method to assist numerical simulation in the optimization of the forging process. Chauhan et al. [42] not only applied Moldflow analysis in the optimization of natural fiber polymeric composite but employed Taguchi design to assist in the optimization. Sharma and Sharma [43] employed the Taguchi design technique in the synthesis and structural analysis of a Cassia galactomannan hydroxypropyl derivative. Abifarin and Ofodu [38] employed the Taguchi design technique to simulate chemical additives and engine parameters for better engine performance efficiency. From the review of the optimization research above, this paper employs the Taguchi technique assisted by GRA to optimize CNC machine conditions for better composite performance. This paper also integrates modeling and interaction as optimization assistance, providing opportunities for material production experts to explore different CNC machine capacities.

### 2.3. Research Gaps Analysis

Having expressed some previous research conducted on the optimization of hybrid composite, it is clear that there is still a gap in the area of optimization modeling, factor interactions, and deeper multi-response optimization analysis in the production of hybrid composite for highly efficient performance in terms of cost-effectiveness and quality of the produced hybrid composite. Hence this study employed Taguchi GRA, modeling, and interaction analysis in the optimization of the halloysite nanotube (HNTs) hybrid composite. The hybrid composite as a case study was selected to fill the many gaps left in hybrid composite development, especially from the study by Pang et al. [21]. As indicated in the literature review (See Section 2.1), several optimization settings have been employed in the machine manufacturing of hybrid composite. However, for the first time, this study added mathematical, graphical modeling and the interaction of CNC machine conditions to the requirements’ singular and multi-response GRA optimization analysis. This paper, as a result, provides a clearer, more substantial, and conclusive CNC machine setting for the manufacturing of hybrid polymer composite. This paper also provides different optimization setting opportunities for material production experts for various CNC machine capacities. Table 1 presents a literature review with research findings and gaps.

## 3. Materials and Methods

### 3.1. Data Curation

**Figure 1:**
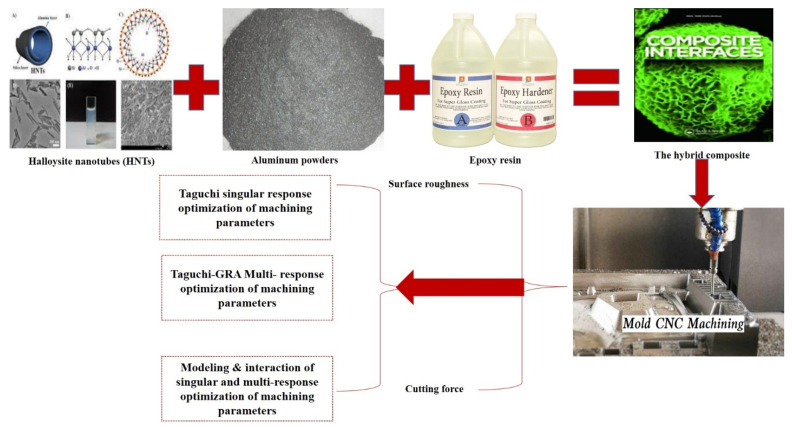
Data curation and design analysis.

As said in the introductory section, this study builds on the work of Pang et al. [21]. Hence, the hybrid composite development, characterizations, and data curation have been presented in the study by Pang et al. [21]. In addition, please refer to the study of Pang et al. [21] for the type of milling machine used and the conditions under which the milling operation was performed. Figure 1 shows how the data were curated for the present analysis.

### 3.2. Design Analysis

Based on the experimental procedures described in Section 3.1, the quality of the hybrid composite in terms of surface roughness and its production cost in terms of cutting force were analyzed using three machining parameters: depth of cut, spindle speed, and feed rate. The breakdown of the design is shown in Table 2.

With the help of the Taguchi design technique, an orthogonal array with nine design runs was employed based on a strategy to have better design efficiency and a reduced laborious nature of the work [46,47]. This was not considered in the study of Pang et al. [21]. Table 3 presents the Taguchi design runs in detail.

### 3.3. Taguchi Design Applicability

The orthogonal arrays of the Taguchi design method were introduced to minimize cost and maximize product quality. The signal-to-noise (S/N) ratio is usually applied to the performance characteristics of the process or product to know the process or product strength and to the degree of the deviation of true values from the desired values. The S/N ratio is computed in the space of logarithmic function as a response mean (signal) versus standard deviation (process or product noise) [22,48]. In this study, the smaller, better S/N ratios (Equation (1)) indicate better product quality and reduced production cost. As small a surface roughness as possible is needed on the manufactured composite because it reflects better surface finishing of the composite, i.e., a very high composite quality. Further, reducing the cutting force of the CNC machine as much as possible is required to reduce the energy consumption because the higher the cutting force, the higher the energy consumption, and vice versa. The reduced energy consumption during the machining of the composite reduces overall production costs. In Equation (1), n is the design run number, and $y_{i}$ is the response value of the ith design run in the table (See Table 2).

(1) $${\frac{s}{N}}_{SIB}=-10\times \log_{10}\left(\frac{1}{n}\sum_{i=1}^{n}y_{i}^{2}\right)$$

### 3.4. Multi-Characteristic Optimization of Machining Parameters with GRA

As the introductory section states, the Taguchi DOE technique can only optimize the processing conditions for singular performance characteristics. This study has a situation of two different aspects; GRA is employed to bring about a conclusive optimization remark without trading off either of the two considered responses, which made the situation less complicated [37,49,50,51]. The below presents the GRA stages on the multi-response optimization.

#### 3.4.1. Grey Relational Generation

In the analysis of the considered data using GRA, the machining parameter function is ignored when there is a situation whereby the standard value and the reference sequence range are high. Moreover, surface roughness and cutting force as responses are not within the same space in this study; hence GRA may generate inaccurate results. Thus, the two responses were preprocessed to normalize the rather untransformed data to compare data in the range of zero to one [37,49,52,53]. The process of normalization is referred to as grey relational generation. Similar to individual response optimization, smaller is better, and analysis was employed for the normalization of the data, as shown in Equation (2):

(2) $$x_{i}\left(k\right)=\frac{{{\max\;y}_{i}\left(k\right)-y}_{i}\left(k\right)}{{\max\;y}_{i}\left(k\right)-\min y_{i}\left(k\right)\;}$$

Note that $x_{i}\left(k\right)$ is the preprocessed data for the ith design run, and $y_{i}\left(k\right)$ is the untransformed response data.

#### 3.4.2. Grey Relational Analysis

After normalization of the untransformed response data, the deviation sequence was computed using Equation (3):

(3) $$\Delta_{oi}\left(k\right)=∥1-x_{i}\left(k\right)∥$$

Note that $\Delta_{oi}\left(k\right)$ and $x_{i\;}\left(k\right)$ reflect the deviation and the normalized sequences, respectively.

Next, the grey relational coefficient (GRC) was computed using Equation (4):

(4) $$\xi_{i}\left(k\right)=\frac{\Delta_{min}+\zeta \Delta_{max}}{\Delta_{oi}\left(k\right)+\zeta \Delta_{max}}$$

Note that $\xi_{i}\left(k\right)$ is the GRC of individual responses (surface roughness and cutting force), which is a function of Δ${}_{min}$ and Δ${}_{max}$, the minimum and the maximum deviations of each response data. ζ represents the distinguishing coefficient (0∼1). A ζ equal to 0.5 was assigned to the two responses, which is the assigned usual value.

Equation (5) shows how to compute grey relational grade (GRG), which is the average of GRC.

(5) $$\gamma_{i}=\frac{1}{n}\sum_{i=1}^{n}\xi_{i}\left(k\right)$$

Note that $\gamma_{i}$ is the GRG, and n is the number of performance characteristics, which is two in this study (surface roughness and cutting force).

### 3.5. Variance Analysis (ANOVA)

To know the significance and the quantitative effect of the machining parameters on the individual response optimization and the multi-response optimization, ANOVA was employed. The F-analysis in ANOVA was employed to determine the degree to which the machining parameters control the analyzed results. Using a 95% confidence level, if ‘Prob > F’ and is less than 0.05, the machining parameters and interactions are reflected to be significant [43]. Additionally, when the F-value is very large, it means that the machining parameter has a very significant effect on the performance characteristic. R${}^{2}$ is the adjusted correlation coefficient that was used to understand the strength of the fitted model, while R${}^{2}$${}_{adj}$ investigates the proportion of disparity clarified exclusively by each machining parameter. Lastly, it can be strongly concluded that the created models perfectly fit the design project well when the values of R${}^{2}$ and R${}^{2}$${}_{adj}$ are high and close to each other [29,43,46].

### 3.6. Regression Modeling and Interaction Analysis

Regression analysis was employed to model the values of surface roughness, cutting force, and GRG at different design runs using Minitab 16 software. In the mathematical modeling generated, A is shown as the depth of cut, B is shown as the spindle speed, and C is shown as the feed rate. Equations (6)–(8) are generated with the regression modeling.

To generate the interaction plot, Origin 19 software was employed. The interaction was used to examine the effect of machining parameters on the performance characteristics (surface roughness, cutting force, and GRG) of the hybrid composite. This was performed to have a holistic view of how machining parameters can be set for different desired responses.

## 4. Results and Discussion

### 4.1. Data Inspection Analysis

Table 4 presents the orthogonal array of the experimental data properties under different machining conditions, supported by Table 3. From the presented results, it is clear that surface roughness and cutting force are not in the same space or even within the same range. This makes it difficult to conclusively conduct multiple optimizations. From data inspection without optimization analysis, one can pinpoint machining parameters for as small as possible surface roughness and cutting force. The third experimental run, having a 0.4 mm depth of cut, 1500 rpm spindle speed, and 60 mmpm feed rate, showed the lowest surface roughness value; hence they are the best machining parameters for a high-quality hybrid composite. However, the fourth experimental run, having a 0.6 mm depth of cut, 500 rpm spindle speed, and 40 mmpm feed rate, showed the smallest cutting force, which made it the best machining parameters for as low as possible cost. For singular performance characteristics, data inspection can be applied for optimization, but it is, however, deficient because it does not consider some other probable combinations of processing parameters outside the presented data. It also does not consider the peculiarity of the data in terms of repeatability. Data inspection optimization leaves the possibility of not having the best optimal settings within the presented data. Significantly, Taguchi’s design optimization integrates this shortfall into the system by considering some other probable combination of processing parameters outside the presented data. It also presents an in-depth data analysis for a substantial conclusion. Hence, Taguchi singular optimization for surface roughness and cutting force is presented in Section 4.2 and Section 4.3, respectively.

### 4.2. Quality Maximization Analysis

#### 4.2.1. Surface Roughness Response Table Analysis

For better quality of the hybrid composite, as small as possible surface roughness is required for smooth better surface finishing. Surface roughness data presented in Table 4 were analyzed with Taguchi, and the response means according to the machining parameters are shown in Table 5. From the table, the optimal settings are obtained based on the smallest data from the three levels presented under each machining parameter. The depth of cut under Level 2 displayed the smallest surface roughness value, the spindle speed under Level 3 displayed the smallest surface roughness value, and the feed rate under Level 1 displayed the smallest surface roughness value. Hence, Table 3 shows the actual machining parameters for the levels identified with the smallest surface roughness value. i.e., 0.6 mm depth of cut, 1500 rpm spindle speed, and 20 mmpm feed rate are the best optimal settings for a high-quality surface finishing. As it was said on the deficiency of data inspection on experimental repeatability, the sixth experimental run gave the optimal settings for the data inspection optimization analysis. Furthermore, it is important to note the significance of each machining parameter on the resultant surface roughness. The statistics reflecting delta is the range of the surface roughness values under each machining parameter. The delta characteristic of the depth of cut reflected the highest value, followed by that of the spindle speed and then that of the feed rate. These characteristics are ranked in descending order. These characteristics showed that the depth of the cut is the most significant machining parameter, followed by spindle speed.

#### 4.2.2. Effect of Machining Parameters on Surface Roughness

**Figure 2:**
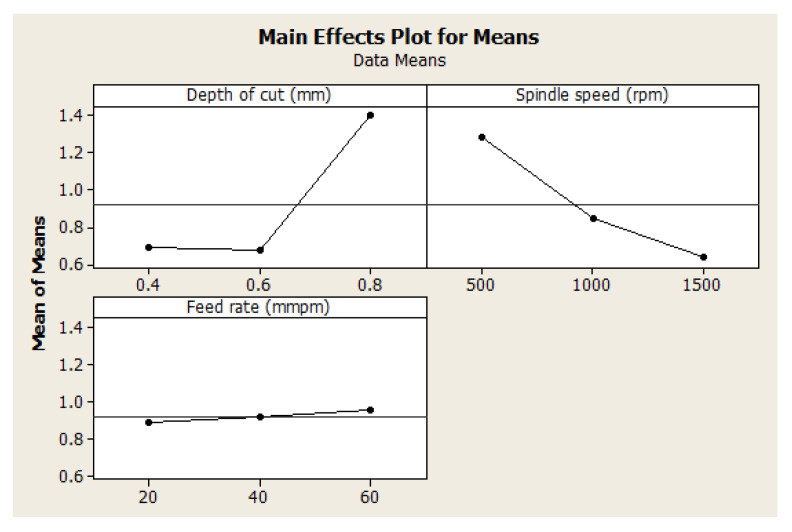
Effect of machining parameters on surface roughness.

The effect of the machining parameters on the surface roughness of the manufactured hybrid composite is presented in Figure 2. It is shown that surface roughness increased with the depth of cut. This observation is not far-fetched from the fact that an increased depth of cut would exact much more energy on the surface of the workpiece. This, in turn, would lead to a rough application of the cutter on the workpiece, thereby leading to a lot of surface roughness. This finding is in agreement with the literature [50,51,54,55,56]. The depth of cut, however, geometrically increased the surface roughness at 0.8 mm depth of cut. This shows that it is better to keep the depth of cut under 0.6 mm. Conversely, an increase in spindle speed reduced the surface roughness in a significant manner, and it is in agreement with the findings by Pathak et al. [50]. However, for the feed rate, an increase in feed rate increased the surface roughness. In the selection of optimal settings for high-quality surface finishing, Figure 2 equally revealed those machining parameters with the smallest surface finishing. It is shown that 0.6 mm depth of cut, 1500 rpm spindle speed, and 20 mmpm feed rate is the optimal machining parameters for high-quality surface finishing. These findings are in absolute agreement with the results displayed in Table 5 (see Section 4.2.1). These findings contradict the findings by Pang et al. [21] because they did not employ the Taguchi design for deeper analysis. It is clear that they only used a direct data plot for optimal setting selection. The findings in Section 3.1 for data inspection optimization revealed the same as those by Pang et al. [21]. Hence, when optimizing for better surface finishing with repeatability and less laborious considerations, 0.6 mm depth of cut, 1500 rpm spindle speed, and 20 mmpm feed rate is the conclusive optimal machining parameters.

**Figure 3:**
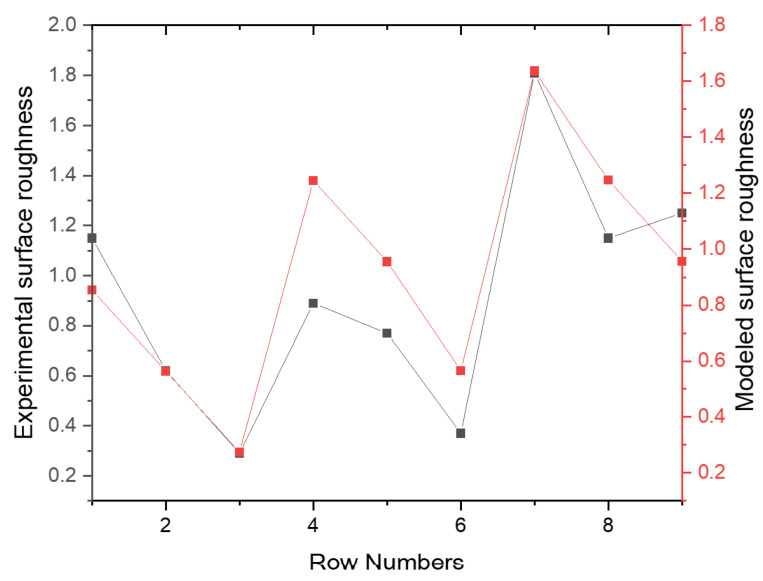
Experimental versus modeled surface roughness.

From the investigation of the optimal machining parameters settings, the variance analysis (ANOVA) in Table 6 revealed the significance of each machining parameter on the surface roughness of the manufactured hybrid composite. Based on the delta statistical characteristics in Table 5, Table 6 also shows the same significant machining parameters. However, Table 6 shows a significant numerical effect of machining parameters on surface roughness on the composite. It is shown here that the depth of cut is the most significant machining parameter, with a contribution of 57.25%, followed by spindle speed, with a contribution of 35.89%. However, it is important to say that the feed rate is invariably insignificant on the resultant surface roughness, having a contribution of 0.37%. Residual error was also less significant, but it suggests that a more careful experiment can be designed to have a lesser influence of error on surface roughness. For the linear fit model, it is revealed that the $R^{2}$ value is high compared to the $R_{adj}^{2}$ value. This explains the contribution of the residual error having a recommendation for a more careful experimental design to have a better-designed fit model. Figure 3 also elucidates the fitness of the model compared to the experimental reality. It is shown that the experimental surface roughness aligns with the modeled surface roughness from regression analysis.

#### 4.2.3. Surface Roughness Modeling

Equation (6) reveals the mathematical modeling of surface roughness with the considered machining parameters using regression analysis. This equation accommodates for the unconsidered machining parameters, i.e., at any point of machining parameters outside the three different level points analyzed, surface roughness can be determined. This helps the designer navigate the available machining parameter settings in the case that the CNC machine designer has limited capacity. It will help the designer to determine the next best machining parameters for better surface finishing.

(6) $$S_{r}=0.427+1.79A-0.000647B+0.00167C$$

**Figure 4:**
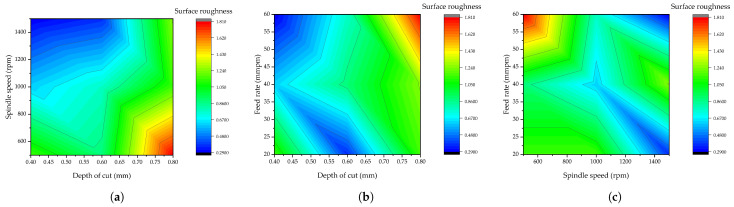
Interaction of the machining parameters on surface roughness. (**a**) Spindle speed versus depth of cut, (**b**) feed rate versus depth of cut, and (**c**) feed rate versus depth of cut.

Additionally, the interaction modeling of the machining parameters employed on the resultant surface roughness is presented in Figure 4. Since as small as possible surface finishing is required, the interaction between the machining parameters revealed a better surface finishing, which is around ≤ 0.4 surface finishing. The range for better surface finishing is displayed with blue colors in all three interaction plots displayed. For spindle speed versus depth of cut interaction, it is revealed that there will be better surface finishing when the spindle speed is greater than or equal to 1200 rpm and when the depth of cut is less than or equal to 0.6 mm. For feed rate versus depth of cut, it is revealed that there will be better surface finishing when the feed rate is greater than or equal to 50 mmpm and also in the range of 20 and 25 mmpm, while the depth of cut should be between 0.4 and 0.5 mm and also between 0.55 and 0.66 mm. Lastly, for feed rate versus spindle speed, it is shown that there will be better surface finishing when the feed rate is between 20 and 30 mmpm and also between 55 and 60 mmpm, while the value of spindle speed should be 1300 rpm or above. This observation can draw out different optimizations depending on the available control of the machining parameters.

### 4.3. Cost Minimization Analysis

#### 4.3.1. Cutting Force Response Table Analysis

As in the case of surface response analysis, the cost of manufacturing the hybrid composite will be low if the production does not require much cutting force, leading to low energy consumption. Hence, as small as possible, a cutting force is required for production cost minimization. The cutting force data are presented in Table 4 and were generated with Taguchi design analysis. The response means of the cutting force according to the machining parameters are shown in Table 7. With the same analogy with surface roughness, the optimal settings are obtained based on the smallest data from the three levels presented under each machining parameter. The results showed that all the machining parameters (depth of cut, spindle speed, and feed rate) exhibited the smallest cutting force at Level 2. Meaning that the optimal settings for the lowest production cost are 0.6 mm depth of cut, 1000 rpm spindle speed, and 40 mmpm feed rate. These findings substantially revealed the deficiency associated with data inspection optimization. The findings revealed an optimal setting outside the experimental run considered in this study. It is also in agreement with the findings by Pang et al. [21] because they considered the entire possible machining parameter combinations, which have defeated the sole aim of the Taguchi design to reduce the laborious nature of the research study. However, the findings in this study sufficiently employed 9 machining parameter combinations with the help of Taguchi design against the 27 machining parameter combinations employed by Pang et al. [21]. The statistics reflecting delta in the range of cutting force under each machining parameter show the significance of the parameters. The higher the delta characteristic, the more significant the machining parameters. The results showed that the delta characteristic of the feed rate reflected the highest value, followed by the depth of cut, and then the spindle speed. These characteristics are ranked in descending order, which means that the feed rate is the most significant machining parameter, followed by the depth of cut and then spindle speed.

#### 4.3.2. Effect of Machining Parameters on Cutting Force

**Figure 5:**
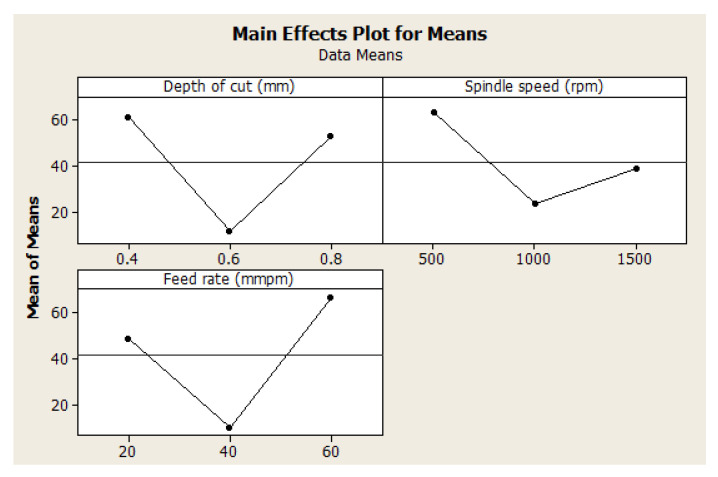
Effect of machining parameters on cutting force.

The effect of machining parameters on the manufacturing hybrid composite’s cutting force is presented in Figure 5. It is shown that there is a gradual increase in cutting force with increases in all machining parameters (depth of cut, spindle speed, and feed rate). From Figure 5, the optimal machining parameters are all at Level 2, supporting the cutting force response analysis (see Section 4.3.1).

**Figure 6:**
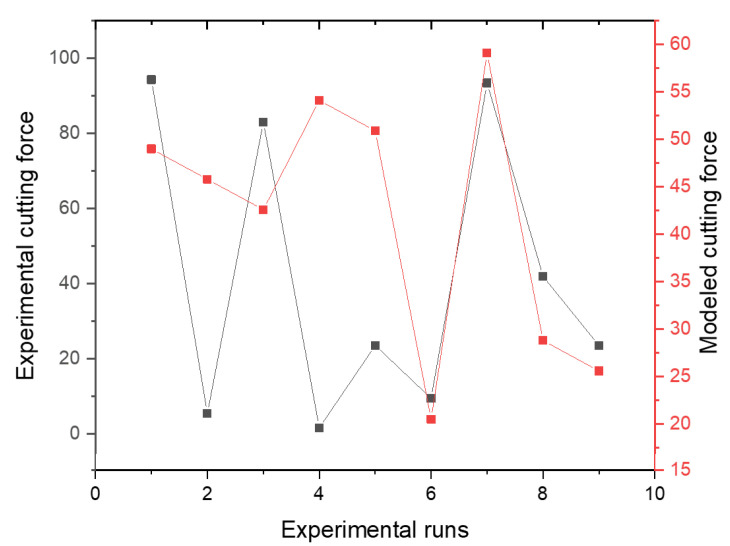
Experimental versus modeled cutting force.

After the analysis of the optimal machining parameter settings for the cutting force, it is important to employ variance analysis (ANOVA) to know the numerical effect of machining parameters on the cutting force holistically. Table 8 revealed the significance of each machining parameter on the cutting force of the manufactured hybrid composite. Based on the delta statistical characteristics in Table 7, Table 8 also shows the same significance of machining parameters on the cutting force of the manufactured composite numerically. Substantially, the feed rate is the most significant machining parameter with a contribution of 42.41%, followed by the depth of cut with a contribution of 35.8%, and then spindle speed with a contribution of 20.23%. The analysis also revealed that residual error is insignificant on cutting force, contributing 1.55%. This shows high efficiency in the experiment’s repeatability and a high confidence level in the experimental data curation. The fit linear model data supported the lesser noise influence on the experimental cutting force, having a high $R^{2}$ close to the $R_{adj}^{2}$ value. Figure 6 also elucidates the fitness of the model compared with the experimental reality graphically. It is shown that the experimental cutting force graph pattern is related to that of the modeled cutting force.

#### 4.3.3. Cutting Force Modeling

Equation (7) reveals the mathematical modeling of cutting force with the machining parameters employed with the help of regression analysis. This equation will help the design engineer to maneuvre the control of the CNC machine at any point of machining parameters outside the three different level points analyzed in the case where the CNC machine is limited in capacity. It will help the designer to determine the next best machining parameters for better cutting force.

(7) $$F_{c}=60.1-19.9A-0.0245B+0.453C$$

**Figure 7:**
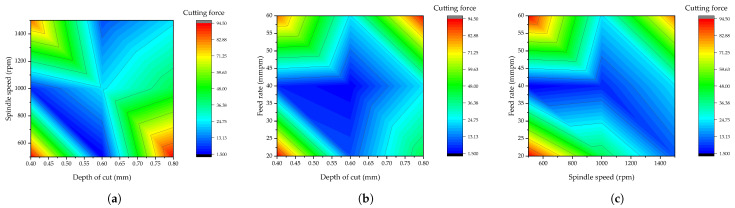
Interaction of the machining parameters on cutting force. (**a**) Spindle speed versus depth of cut, (**b**) feed rate versus depth of cut, and (**c**) feed rate versus depth of cut.

Furthermore, the interaction modeling of the machining parameters employed on the resultant cutting force is shown in Figure 7. As required, a smaller value of cutting force is paramount; hence the interaction between machining parameters revealed a better cutting force of around ≤12.12. The range is also displayed in blue in all three interaction plots. For spindle speed versus depth of cut interaction, it is revealed that there will be a smaller cutting force when the spindle speed is in the range of 900 and 1500 rpm and when the depth of cut is between 0.4 and 0.66 mm. For feed rate versus depth of cut, it is revealed that there will be a lesser cutting force when the feed rate is between 35 and 45 mmpm, and the depth of cut should be between 0.4 and 0.66 mm. Lastly, for feed rate versus spindle speed, it is shown that there will be a lesser cutting force when the feed rate is between 35 and 45 mmpm and at different values of spindle speed, depending on the control level of the CNC machine. Similar to the findings in Section 4.2.3, other optimizations can be drawn out with different CNC machines.

### 4.4. Multi-Performance Optimization Analysis

From the analysis of singular optimization of the two performance characteristics (surface roughness and cutting force), it is clear that the two characteristics have their optimal machining parameters at different conditions. Since the two characteristics are important to be considered in the conclusive selection of machining parameters for better production of the hybrid composite, then it becomes more complicated to choose either of the optimal settings. This drawback was the major problem and gap identified in several studies, including the study by Pang et al. [21]. Hence, GRA was employed in this study to address the complication in the selection of conclusive optimal machining parameter settings with the consideration of the two performance characteristics holistically. Grey relational grade (GRG), as a combination of both the surface roughness and the cutting force, was computed according to Equations (2)–(5). The GRG values were then analyzed with Taguchi for accurate analysis. Below shows the conclusive findings of the optimal machining parameter settings in the manufacturing of hybrid composite.

#### 4.4.1. Grey Relational Grade (GRG) Response Table Analysis

From a holistic point of view, better manufacturing of hybrid composite requires as high as possible GRG value. Therefore, a larger is better design analysis was employed in the Taguchi design. The mean GRG response values are presented in Table 9 according to their respective machining parameters. From the table, the optimal settings are obtained based on the highest GRG value from the three levels presented under each machining parameter. The results show that the depth of cut has the highest GRG value at Level 2 (0.6 mm depth of cut), spindle speed has the highest GRG value at Level 3 (1500 rpm depth of cut), while the feed rate has the highest GRG value at Level 2 (40 mmpm). These findings showed that it is at those levels that the manufacturing of the hybrid composite will be of high quality with a low cost of production; they are the substantial optimal machining parameter settings for multi-performance characteristics (surface roughness and cutting force). These machining parameter settings are not within the nine experimental runs employed in this study, which shows the high efficiency of the GRA design. The statistics’ delta of the GRG values under each machining condition reveals the level of significance of the parameter on GRG values, and it is the range of GRG values under each machining parameter. The delta statistics revealed that the depth of cut is the most significant factor, followed by the spindle speed and then the feed rate. These characteristics are ranked in descending order.

#### 4.4.2. Effect of Machining Parameters on GRG

**Figure 8:**
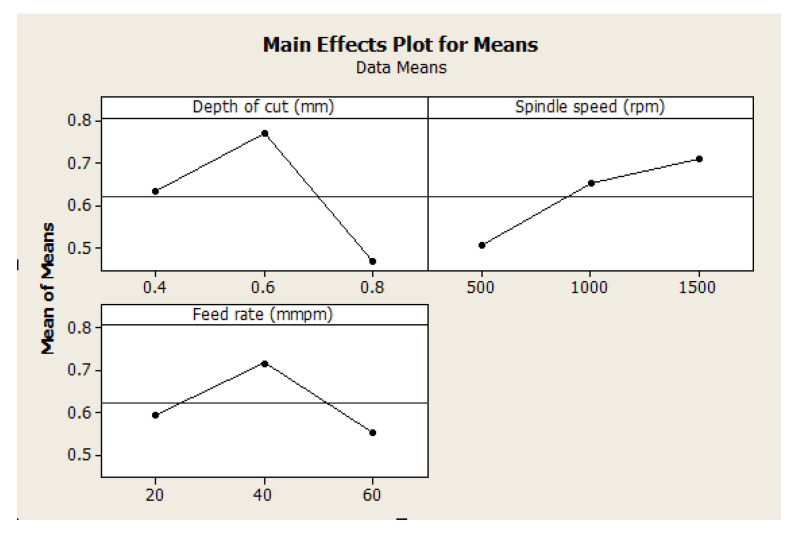
Effect of machining parameters on GRG.

The effect of machining parameters on GRG values of the manufactured hybrid composite has been presented in Figure 8. It is shown that there is a gradual increase in GRG value with an increase in depth of cut and feed rate but a proportional increase with an increase in spindle speed. This means that spindle speed improves the overall efficiency of the production in terms of surface finishing with low production costs. This is in agreement with the studies by Muhammad et al. [54], Usca et al. [55], and BM et al. [56]. As analyzed under response GRG analysis, Figure 8 shows the same optimal machining parameter settings for high-quality products with low cost.

**Figure 9:**
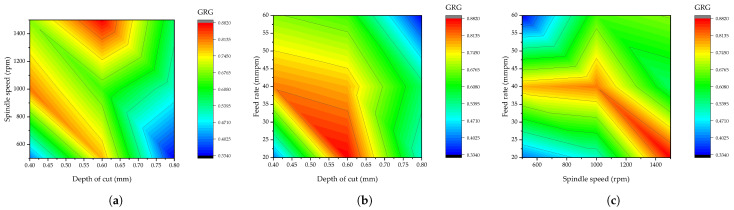
Interaction of the machining parameters on GRG. (**a**) Spindle speed versus depth of cut, (**b**) feed rate versus depth of cut, and (**c**) feed rate versus depth of cut.

As discussed for singular response optimization, variance analysis (ANOVA) in Table 10 revealed the significance of each machining parameter on the GRG value of the manufactured hybrid composite. These findings agree with delta statistics, but variance analysis numerically showed the significance level. The results showed that the depth of cut is the most significant factor, with a contribution of 49.12%, followed by spindle speed, with a contribution of 23.43%, and then feed rate, with a contribution of 15.4%. Here, the findings revealed that residual error also has a significant contribution (12.06%), which shows the influence of noise in the experimental data (surface roughness and cutting force) curation. The linear fit model has a relatively small value of $R^{2}$; it is far from the $R_{adj}^{2}$ value. This validates the influence of noise identified in the design model. Figure 9 also elucidates the non-fitness of the predicted model compared to the experimental one. These findings suggest that researchers and design engineers have careful design considerations and conditions when dealing with CNC machines in manufacturing hybrid composites.

#### 4.4.3. GRG Modeling

Equation (8) is the mathematical model of the GRG values with the three machining parameters using regression analysis. This equation accommodates for the unconsidered machining parameters, i.e., at any point of machining parameters outside the three different level points analyzed; the GRG value can be determined. This helps researchers or design engineers to navigate the available machining parameter settings in the case where the CNC machine has limited capacity.

(8) GRG=0.708−0.414A+0.000202−0.00102C

Moreover, the interaction modeling of the machining parameters employed on the resultant GRG value is presented in Figure 9. In this case, a high GRG value is required for a better result; hence, the analysis is performed to have a GRG value greater than or equal to 0.8. The range for a higher GRG value (better multi-response characteristic) is displayed in red for all three interaction plots displayed. For spindle speed versus depth of cut interaction, it is revealed that there will be a higher GRG value when the spindle speed is greater than 1400 rpm and when the depth of cut is between 0.56 and 0.66 mm. For feed rate versus depth of cut, it is revealed that there will be a higher GRG value when the feed rate is between 35 and 45 mmpm, and the depth of cut is between 0.55 and 0.65 mm. Lastly, for feed rate versus spindle speed, it is shown that there will be a higher GRG value when the feed rate is around 40 mmpm and when the spindle speed is around 1400 rpm or above. This helps make an adjustment with CNC machines for the efficient production of hybrid composites based on the peculiarity of the available machining conditions.

## 5. Conclusions

This study has substantially employed statistical tools with the help of GRA to bridge the gap in the investigation of quality and cost optimization of manufactured halloysite nanotube hybrid composites. The results revealed that traditional data inspections of singular optimizations of surface roughness and cutting force are not enough compared to the Taguchi design technique. Due to complications encountered for multi-response optimization, it was shown that GRA was able to solve the optimization complication by generating 0.6 mm depth of cut, 1500 rpm spindle speed, and 40 mmpm feed rate as the main optimal settings for the production of high-quality hybrid composites with a relatively low production cost. The mathematical and interaction modeling for surface roughness, cutting force, and GRG accommodates the employment of different CNC machines in the manufacturing of hybrid composites, which will assist researchers or design engineers in determining different optimizations based on the peculiarity of the employed machine. The variance analysis and delta statistical characteristics revealed the significance level of the machining parameters employed for the singular and the multi-response optimization analysis. The results revealed that the depth of cut is the most significant machining parameter, having a contribution of 49.12%, followed by spindle speed with a contribution of 23.43%, then feed rate with a contribution of 15.4%. It is, however, important to state that residual error influenced the resultant multi-performance characteristic with a contribution of 12.06%. The influence of residual errors for all the optimization analyses was substantially validated with a linear fit model and model graphs.

The mathematical modeling and the multi-performance optimization findings are recommended for researchers and production engineers who manufacture halloysite nanotube hybrid composites with CNC machines. It is of great interest to say that the results from this study allow the adjustment of optimal machining parameter settings depending on the available type and capacity of the CNC machine. It is recommended for researchers in this field to consider modeling analysis with singular and multi-response optimization techniques when conducting experiments on CNC machine material manufacturing. This paper is limited to simulation only. The simulation was made from the physical experimental data presented in the study by Pang et al. [21]. Since this study has been able to generate a general model beyond the scope of the machining conditions considered in the experimental work by Pang et al. [21], it is recommended that a physical machining assessment beyond the study by Pang et al. [21] using the designs and modeling generated in this study should be carried out in the future. 

## Figures and Tables

**Table 1:** Summary of research gaps in the literature.

Researchers	Methods	Material Types	Applications	Limitations
Pang et al. [21]	Taguchi technique	Halloysite nanotube hybrid composite	Machine parameters	Inefficient singular optimization with no variance analysis
La et al. [44]	Taguchi grey relational analysis technique	Aluminum metallic hybrid composite	Machine parameters	No interaction and modeling of machine parameters
Kalantari et al. [25]	Pareto optimal solutions technique	Epoxy hybrid composite	Density and cost parameters	Inefficient singular optimization
Rao et al. [26]	Taguchi and elitist non-dominated sorting genetic algorithm technique	Epoxy hybrid composite	Jet machine parameters	No modeling for general conclusion
An et al. [27]	Multipoint approximate functions and genetic algorithm technique	Laminated hybrid composite	Fundamental frequency and material cost parameters	Inefficient singular optimization with no variance analysis
Ma et al. [28]	Discrete material technique	Hybrid composite	Framework parameters	Inefficient singular optimization
Beylergil [29]	Multi-objective genetic algorithm technique	Glass fiber hybrid composite	Fiber properties	Inadequate for other salient parameters such as material cost
Kayaroganam et al. [30]	Non-dominated sorting genetic algorithm and statistical analysis technique	Metal hybrid composite	Drilling parameters	No interaction and modeling of drilling parameters
Kumar et al. [31]	Analytical hierarchy process and genetic algorithm technique	Aluminum metal matrix hybrid composite	Machining parameters	No interaction and modeling of machine parameters
Kandasamy et al. [32]	Taguchi design technique	Aluminum hybrid composite	Erosion rate parameter	Inefficient singular optimization
Peng et al. [33]	Adaptive non-dominated sorting genetic algorithm technique	Laminated hybrid composite	Production parameters	No interaction and modeling of production parameters
González et al. [34]	Traditional experimental technique	Grinding flank tools	Super abrasive integral rotary components	No optimization
Kondayya and Krishna [35]	Integrated evolutionary technique	Materials simulation	CNC end-milling parameters	No analysis of cutting force and surface roughness
Ribeiro and Lopes [36]	Taguchi design technique	Hardened steel block (steel 1.2738) with tungsten carbide-coated tools	End-milling parameters	Inefficient singular optimization
Achuthamenon Sylajakumari et al. [37]	Taguchi grey relational analysis technique	Co-continuous composite	Wear parameters	No interaction and modeling of wear parameters
Abifarin et al. [45]	Taguchi grey relational analysis technique	3D chitosan/PLA composite	Fabrication parameters	No interaction and modeling of fabrication parameters

**Table 2:** Machining parameters and their application levels.

Machining Parameters	Depth of Cut (mm)	Spindle Speed (rpm)	Feed Rate (mmpm)
Level 1	0.4	500	20
Level 2	0.6	1000	40
Level 3	0.8	1500	60

**Table 3:** Taguchi design strategy.

Design Runs	Depth of Cut (mm)	Spindle Speed (rpm)	Feed Rate (mmpm)
1	0.4	500	20
2	0.4	1000	40
3	0.4	1500	60
4	0.6	500	40
5	0.6	1000	60
6	0.6	1500	20
7	0.8	500	60
8	0.8	1000	20
9	0.8	1500	40

**Table 4:** Experimental data.

Design Runs	Surface Roughness (µm)	Cutting Force (N)
1	1.15	94.31
2	0.62	5.45
3	0.29	83.02
4	0.89	1.63
5	0.77	23.6
6	0.37	9.46
7	1.81	93.45
8	1.15	41.94
9	1.25	23.51

**Table 5:** Surface roughness response table (smaller is better).

Factors	Depth of Cut	Spindle Speed	Feed Rate
Level 1	0.6867	1.2833	0.89
Level 2	0.6767	0.8467	0.92
Level 3	1.4033	0.6367	0.9567
Delta	0.7267	0.6467	0.0667
Rank	1	2	3

**Table 6:** The significance of machining parameters on cutting force.

Factors	DOF	Adj SS	Adj MS	F	Contribution (%)	Remark
Depth of cut (mm)	2	1.04176	0.520878	8.82	57.25	Most significant
Spindle speed (rpm)	2	0.65296	0.326478	5.53	35.89	Significant
Feed rate (mmpm)	2	0.00669	0.003344	0.06	0.37	Insignificant
Residual error	2	0.11816	0.059078		6.49	Less significant
Total	8	1.81956	0.909778	S = 0.2431	$R^{2}$ = 93.5%	$R_{adj}^{2}$ = 74.0%

**Table 7:** Cutting force response table (smaller is better).

Factors	Depth of Cut	Spindle Speed	Feed Rate
Level 1	60.93	63.13	48.57
Level 2	11.56	23.66	10.2
Level 3	52.97	38.66	66.69
Delta	49.36	39.47	56.49
Rank	2	3	1

**Table 8:** The significance of machining parameters on cutting force.

Factors	DOF	Adj SS	Adj MS	F	Contribution (%)	Remark
Depth of cut (mm)	2	4214.3	2107.17	23.05	35.8	Significant
Spindle speed (rpm)	2	2381.2	1190.62	13.03	20.23	Significant
Feed rate (mmpm)	2	4992.3	2496.17	27.31	42.41	Most significant
Residual error	2	182.8	91.4		1.55	Insignificant
Total	8	11,770.7	5885.36	S = 9.560	*R*${}^{2}$ = 98.4%	$R_{Adj}^{2}$ = 93.8%

**Table 9:** GRG response table (higher is better).

Factors	Depth of Cut	Spindle Speed	Feed Rate
Level 1	0.6311	0.505	0.5944
Level 2	0.7684	0.6527	0.7168
Level 3	0.4656	0.7074	0.5538
Delta	0.3028	0.2024	0.163
Rank	1	2	3

**Table 10:** The significance of machining parameters on GRG.

Factors	DOF	Adj SS	Adj MS	F	Contribution (%)	Remark
Depth of cut (mm)	2	0.13788	0.06894	4.08	49.12	Most significant
Spindle speed (rpm)	2	0.06575	0.03288	1.94	23.43	Significant
Feed rate (mmpm)	2	0.04322	0.02161	1.28	15.4	Significant
Residual error	2	0.03383	0.01692		12.06	Significant
Total	8	0.28068	0.33484	S = 0.1301	$R^{2}=87.9\%$	$R_{Adj}^{2}=51.8\%$

## Data Availability

Experimental data is provided by Pang et al. [21].
